# Translation, cultural adaptation, and validation of a lipedema symptoms questionnaire

**DOI:** 10.1590/1677-5449.200049

**Published:** 2020-08-31

**Authors:** Alexandre Campos Moraes Amato, Fernando Campos Moraes Amato, Daniel Augusto Benitti, Ricardo Virgínio dos Santos

**Affiliations:** 1 Universidade de Santo Amaro – UNISA, Disciplina de Cirurgia Vascular, São Paulo, SP, Brasil.; 2 Amato - Instituto de Medicina Avançada, São Paulo, SP, Brasil.; 3 Medical Valens Center, Campinas, SP, Brasil.

**Keywords:** questionnaires, translation, cross-cultural comparison, lipedema, obesity, lymphedema

## Abstract

**Background:**

Lipedema is characterized as abnormal bilateral deposition of fat in the buttocks and legs that may be accompanied by orthostatic edema in women. A questionnaire for assessment of lipedema symptoms has previously been published in German and English to assess treatment progress.

**Objectives:**

To translate, culturally adapt, and validate the lipedema symptoms assessment questionnaire for Brazilian Portuguese.

**Methods:**

The process involved three translations and two back-translations performed by independent translators, followed by construction of a consensus version and adaptation according patients’ comprehension. The questionnaire was converted into a digital version and administered to 56 volunteers and then administered to 154 patients from a vascular clinic and correlated with volume determined by segmental bioimpedance.

**Results:**

The 20 pre-test patients were female and at least 90% of the interviewees were able to understand the questions in the final phase. Volunteers had a 96.4% rate of comprehension of the digital online version and a mean completion time of 4 minutes. In 154 patients, limb volume was positively correlated with intensity of symptoms, as assessed by the translated questionnaire, and 3 out of 15 questions were weakly correlated with educational level.

**Conclusions:**

The translated and culturally adapted Brazilian Portuguese version of the lipedema symptoms assessment questionnaire (QuASiL) is a practical tool that is easy and quick to administer and can be used in our population. Additional studies are still needed to assess the instrument’s sensitivity as an aid for diagnosis of lipedema.

## INTRODUCTION

Lipedema was described for the first time in 1940 by doctors Edgar Van Nuys Allen and Edgar Alphonso Hines Jr., at the Mayo Clinic, who characterized it as abnormal bilateral deposition of fat in the gluteus and legs, which may be accompanied by orthostatic edema^1^^,^^2^ in women. Even today, the pathophysiology and epidemiology of lipedema are poorly understood, but it has been suggested that it has a genetic element and is influenced by hormones in cycles of inflammatory symptoms.^3^ Although it is a distinct entity, these factors lead to it often being confused with more frequently diagnosed diseases, such as obesity and lymphedema.^4^^,^^5^ Diagnosis is clinical and is typically defined by the symmetrical disproportion of fat build-up in the lower limbs with complaints of orthostatic edema,^4^ which is frequently accompanied by feelings of heaviness, tiredness, tension, or hard to define pain, which may be constant or provoked by touching the most painful areas and has variable intensity and does not radiate. The feet are spared from the increase in size, except in the advanced stage of lipolymphedema, in which edema of the feet occurs secondary to lymphatic insufficiency, which is not present in earlier stages.^6^^,^^7^ This foot-sparing edema is an important sign for differentiating lipedema from common obesity. The upper body (trunk) is also spared until the most advanced disease stages, although there are some atypical lipedema subtypes in which the expected pattern of lower limb fat build-up can vary.^8^^,^^9^ The areas affected by lipedema often suffer hematoma, pain, and increased sensitivity, which are accompanied by systemic complaints of exhaustion and reduced physical fitness and muscle strength. Onset of symptoms is frequently during puberty or young adulthood, although in some patients it may begin later.^4^ Conservative estimates of the prevalence of lipedema in the general population vary from 0.06 to 10%.^4^

A questionnaire specifically for lipedema (with no title) was developed in Germany for preoperative and postoperative assessment of lipedema symptoms and published by Rapprich et al. in both German^10^ and English.^11^ The questionnaire was based on a quality of life questionnaire for patients with lymphatic diseases^12^ and adapted to include fifteen self-assessed criteria rated on an analog scale from 0 to 10. The questionnaire is based on quality of life assessment. It can be considered a lipedema symptoms scale and has not been validated for use as a diagnostic criterion.

The original questionnaire is interpreted using the intensity of symptoms rated on a visual analog scale and has a total score ranging from 0 to 150, where 0 represents no symptomatic complaints and 150 indicates all symptoms with the highest impact on quality of life. The rarity of objective instruments available for assessment of lipedema in Brazil and worldwide and the fact that quality of life data are important for selection and interpretation of clinical course justify conducting this study with the objective of translating the questionnaire into Portuguese, culturally adapting it for the Brazilian population, and validating it in clinical practice.

## METHOD

This study followed the guidelines set out in National Health Council resolution 466/12 on research involving human beings. It also complies with the Helsinki Declaration and was approved by the Plataforma Brasil Research Ethics Committee, under protocol number 09590919.6.0000.0081.

### Translation and cultural adaptation method

**Figure 1:**
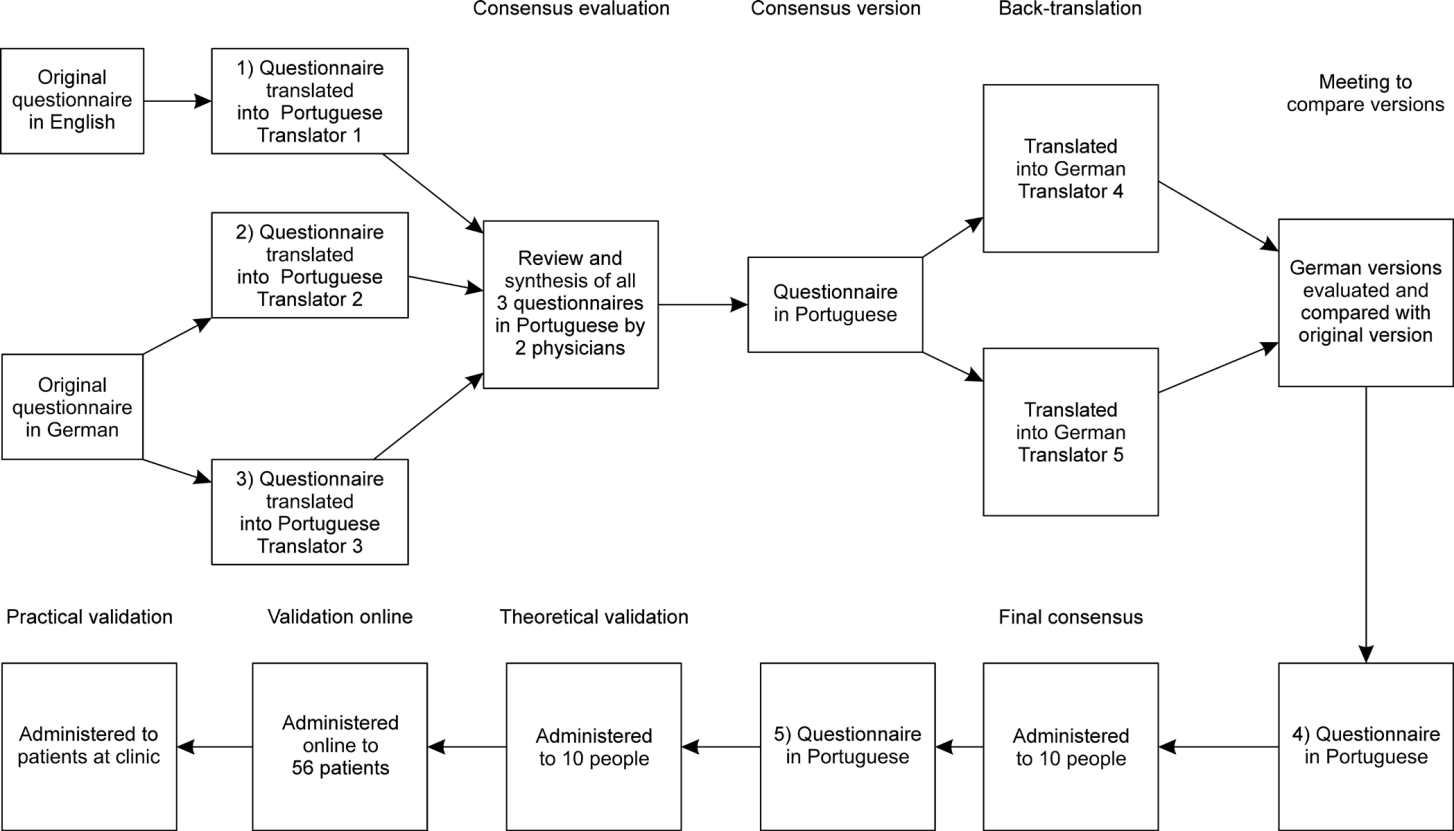
Flow diagram illustrating translation, adaptation, and validation.

The process of achieving linguistic equivalence began by contacting the original author to define concepts and obtain authorization for use. Translation and cultural adaptation of the instrument were conducted according to existing guidelines,^13^^,^^14^ and consisted of translation, back-translation, review by an expert panel, and cultural adaptation (Figure 1).

The items from the English version of the original questionnaire were initially translated by an independent Brazilian healthcare professional with extensive knowledge of the English language who was aware of the research objectives. The questionnaire in German was translated separately by two translators with extensive knowledge of German, who were also aware of the research objectives. Two translations of the original German version were prepared because the original author is German. Emphasis was put on the importance of performing a conceptual translation rather than a literal translation. This stage resulted in translations 1 to 3 in Brazilian Portuguese. Translations 1 to 3 were then evaluated and synthesized to produced a single consensus version by two physicians who were aware of the research objectives and are experts in lipedema. The consensus version was translated into German by two independent, native, professors of German who had not taken part in the previous stage and were not aware of the objectives nor of the concepts dealt with in the questionnaire. Next, the original instrument was compared with the two new versions in German. An expert panel made up of two physicians documented and analyzed discrepancies. Some verb tenses and sentences in Portuguese were rewritten until a consensus was achieved, resulting in version 4 in Portuguese.

Portuguese version 4 of the questionnaire was administered to a group of 10 people to assess their level of comprehension of the questions, and items that were poorly understood were identified. The expert panel then evaluated these items and adapted them to transmit the same concepts, but in a manner that was easier to understand, even using suggestions made by the respondents themselves and attempting not to change the structure or the assessment properties of these items, thereby arriving at version 5 of the questionnaire. This version 5 (Table 1) was administered to a different group of 10 people, selected at random at the same clinic, and its cultural equivalence was tested again until all items were comprehensible to 90% of interviewees.

**Table 1:** Final Brazilian Portuguese Version of the Lipedema Symptoms Assessment Questionnaire.

Questionário de Avaliação Sintomática do Lipedema (QuASiL)											
Nome:						Data:					
Gradue seus sintomas de 0 (não) a 10 (muito). Se o critério for variável, selecione a intensidade máxima que você sente.											
	0	1	2	3	4	5	6	7	8	9	10
As áreas afetadas são dolorosas? (0 não / 10 muito)											
As áreas afetadas são sensíveis ao toque ou à pressão? (0 não / 10 muito)											
Você tende a ter manchas roxas facilmente e frequentes nas pernas? (Hematomas, equimoses) (0 não / 10 muito)											
Você sente “pressão” ou “tensão” nas pernas? (0 não / 10 muito)											
Sente as pernas “quentes” ou sensação de “queimação”? (0 não / 10 muito)											
Sente suas pernas frias? (0 não / 10 muito)											
Tem câimbras musculares? (0 não / 10 muito frequente)											
Sente peso nas pernas? (0 não / 10 muito)											
Sente cansaço nas pernas? (0 não / 10 muito)											
Sente inchaço nas pernas? (0 não / 10 muito)											
Tem “irritações” na pele? (0 não / 10 muita)											
Sente coceira? (0 não / 10 muita)											
Tem dificuldade para caminhar? Alguma limitação de movimento? (0 não / 10 gravemente)											
Como a condição afeta sua qualidade de vida? (0 nada / 10 gravemente)											
Está satisfeita com a aparência das pernas? (Atenção: 0 muito satisfeita / 10 insatisfeita)											

The original German and English versions of the questionnaire are available in Rapprich et al.^10^ and Rapprich et al.^11^, respectively.

Version 5 of the questionnaire was converted into an on-line digital version using secure and appropriate software for development and analysis of questionnaires (SurveyMonkey, San Mateo, California) and was then administered to 56 volunteers who may or may not have been diagnosed with lipedema.

### Administration of questionnaires

Questionnaires were administered individually, from June to September of 2019, after patients had been approached before medical consultations and the objectives and content of the questionnaire had been explained to them. When a person met the inclusion criteria for the study and had time to answer the questionnaires immediately, they were invited to take part and requested to provide consent.

The sampling technique was non-probabilistic, by convenience, and participants were recruited at a Lymphedema and Angiodysplasia Clinic. Patients then had their histories taken and physical examinations, unrelated to the study, and underwent a bioimpedance test, followed by application of inclusion and exclusion criteria.

Participants were women, over the age of 18 years, seen for any complaint whatsoever. Males were excluded and so were people who did not sign the consent form, who had severe arterial or venous conditions, or who were unable to speak or understand Portuguese.

During the on-line phase of questionnaire validation, volunteers from a group specifically of lipedema patients agreed to answer the digital version of the questionnaire, in March 2020, without external help and filling in all details at will.

The primary objective of this study was translation and cultural adaptation of the questionnaire. A secondary objective was to evaluate correlations between the symptoms score and indirect segmental bioimpedance variables.

### Bioimpedance

Segmental body composition analysis was conducted using a multispectrum bioimpedance digital balance that measures the body’s resistance and reactance (Tanita, BC-601, Illinois, United States). The measurements obtained from the bioimpedance scale [height, weight, body mass index (BMI)] were automatically copied to a dedicated chart and other variables were input using software developed especially for this task, which was used to calculate the volumes of the right lower limb (RLL) and left lower limb (LLL), individually, assuming a fat density of 0.9196 g/mL and a muscle density of 1.06 g/mL.^15^^-^17

### Statistical analysis

The statistical analysis was performed after the consistency of data was checked manually. The statistical method chosen was descriptive frequencies. Correlations between variables on the questionnaire were assessed using Spearman correlation coefficients and the Shapiro-Wilk test. Relationships between limb volume and intensity of symptoms on the questionnaire were assessed using Pearson’s correlation coefficients. Statistical analyses were performed using Student’s *t* test, the Kolmogorov-Smirnov test of uniformity, and the Mann-Whitney test. For the correlations, we adopted a statistical significance level of 0.05%. The software used for data analysis was Excel (Microsoft, Redmond, Washington, EUA) and Wizard 1.9.40 (Evan Miller, Chicago, IL, EUA).

## RESULTS

Twenty people took part in the cultural adaptation assessment. None of the questions were considered non-applicable. The questions were understood by at least 90% of the interviewees and were modified and re-administered until all items achieved a comprehension level exceeding 90%. The final version (number 5), administered on-line to the volunteer population (Table 2) achieved overall comprehension of 96.4% in the study population, with a mean completion time of 4 minutes. There was a moderate negative correlation between the item “Do your legs feel heavy?” and educational level (Spearman ρ -0.316, p = 0.018), a weak negative correlation between the item “Do your legs feel tired?” and educational level (Spearman ρ -0.292, p = 0.029) and a moderate negative correlation between the item “Are you satisfied with the appearance of your legs?” and educational level (Spearman ρ -0.309, p = 0.02). The total score was not normally or uniformly distributed (Shapiro-Wilk z = 2.688, p = 0.004; Kolmogorov-Smirnov D = 0.35, p < 0.001) and was not correlated with weight, BMI, or even educational level, but did have a weak negative correlation with age (Pearson r = -0.280, p = 0.037). A colored scale was added to the questionnaire to make it easier to understand the intensity rating scale.

**Table 2:** Characteristics of the study population used for on-line validation of version 5.

Patient characteristics	**Mean (minimum–maximum or 95%CI)**
Volunteers	56
Age	38.69 (22–67)
Weight	79.46 (50–125)
Height	1.62 (1.5–1.76)
BMI	30.10 (20.81–45.91)
Prior diagnosis of lipedema	71.4% (95%CI 58.5–81.6)
Prior diagnosis of varicose veins	73.2% (95%CI 60.4–83)
Educational level	
Higher education completed	35.7%
Post-graduate degree	35.7%
Secondary education completed	12.5%
Higher education, started but not finished	12.5%
Technical college	3.6%
Understood the entire questionnaire	96.4% (95%CI 87.9–99)
Suggested changes	None
Demographics (home state)	
São Paulo	60%
Paraná	14.5%
Minas Gerais	7.3%
Rio de Janeiro	5.5%
Mato Grosso do Sul	3.6%
Rio Grande do Sul	3.6%
Santa Catarina	3.6%
Rio Grande do Norte	1.8%

CI = confidence interval; BMI = body mass index.

The sample comprised 154 patients who were approached at a Lymphedema and Angiodysplasia Clinic (Table 3), regardless of their diagnosis, underwent bioimpedance with segmental body composition analysis, and answered a lipedema symptoms assessment questionnaire.

**Table 3:** Characteristics of the study population used for practical validation.

**Patient characteristics**	**Mean (minimum–maximum)**
Patients	154
Age	43.74 years (19–79)
Height	163.86 cm (139–183)
Weight	79.612 kg (52.1–130.3)
BMI	29.657 kg/cm^2^ (20.6–45.6)
RLL volume	13,627.22 cm^3^ (9,051.57–24,819.61)
LLL volume	13,448.379 cm^3^ (8,885.915–24,757.54)
Symptoms score	79.091 (29–148)

BMI: body mass index; RLL: right lower limb; LLL: left lower limb.

**Figure 2:**
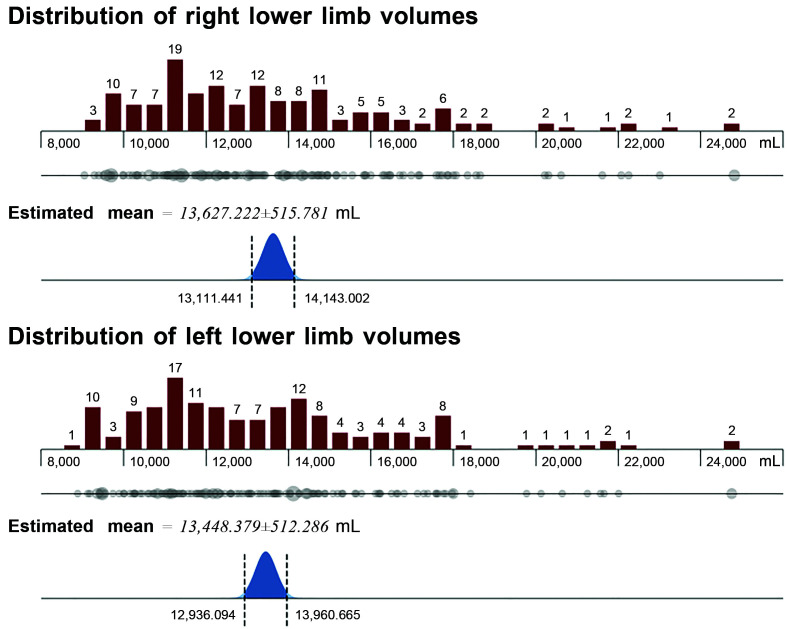
Distribution of lower limb volumes (mL) and estimated means.

The mean and median volumes of left and right limbs were similar (test *t*, p = 0.627; Mann-Whitney test, p = 0.543) and neither variable was uniformly distributed when analyzed individually with the Kolmogorov-Smirnov test (p < 0.001) (Figure 2).

**Figure 3:**
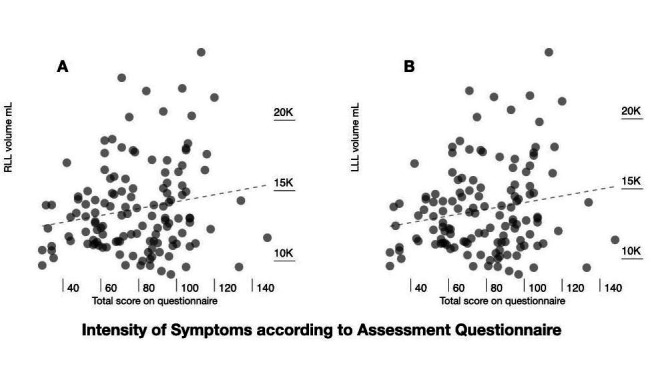
Intensity of symptoms according to assessment questionnaire (total scores) plotted against volumes (mL) of right lower limb (RLL) and left lower limb (LLL).

The RLL volume had a weak positive correlation with the intensity of symptoms assessed by the translated questionnaire (Pearson correlation coefficient r = 0.186, p = 0.034) and LLL volume also had a weak positive correlation with intensity of symptoms (Pearson correlation coefficient r = 0.183, p = 0.037) (Figure 3).

## DISCUSSION

There is a great deal of confusion surrounding lipedema and considerable underdiagnosis because of the lack of a definitive and simple laboratory test or genetic test, combined with a lack of familiarity among physicians with the diagnostic criteria for lipedema.^18^ Lipedema is masked by other conditions and comorbidities, such as lymphedema, physiological disproportionate body shape, lipohypertrophy, and gynecoid obesity, which can contribute to this confusion and lead to wrong diagnosis or underdiagnosis.^19^ Wrong diagnosis of patients with lipedema is of concern, because it delays treatment of the disease, allowing it to develop.^18^ Up to 50% of patients with lipedema are also overweight or obese and the combination of comorbidities makes diagnosis difficult, but does not prevent it, since there are important differences between care for patients with common obesity and those with lipedema.^19^

The inflammatory symptoms of lipedema determine patients’ quality of life,^20^ and patients may have periods of improvement along their lives. Currently, there is no way of monitoring symptomatic development, improvement, or deterioration of lipedema using laboratory tests or imaging exams. Therefore, this questionnaire constitutes a useful tool for assessing the degree of symptomatic compromise the patient is experiencing and for monitoring later development, by comparing patients with themselves.

Measuring the influence that lipedema has on quality of life is still a difficult task because symptoms and complaints are subjective and are confused with other diseases that are very common in the vascular clinic, such as chronic venous insufficiency, obesity, and lymphedema. Although the subject has received greater attention over recent years and there are already several generic and specific instruments for quality of life assessment, the majority of these instruments only assess the differential diagnoses, such as venous insufficiency, obesity, and lymphedema. It is therefore important to develop and validate instruments that can be used to evaluate the impact of lipedema on quality of life and, if possible, aid in arriving at a definitive diagnosis.

After the process of translation, review, and back-translation of the questionnaire, followed by administration to a different population, the final version proved to be highly comprehensible for the study population (96.4%), with weak or moderate correlations with educational level for just 3 items. A correlation does not indicate causality.^21^ We should point out that the scale for the item “Are you satisfied with the appearance of your legs?” has an inverse scale, to facilitate both comprehension and standardization of the questionnaire. Therefore, those with a lower educational level were more unsatisfied with the appearance of their legs. There were no words that the sample could not understand. The inverse correlation between total symptom score and age may imply that symptoms improve or simply that patients’ acceptance of them increases.

The segmental bioimpedance method employed is easy to use and has high reproducibility, although factors such as the subject’s position, the position of the handles, the ambient temperature, hydration level, and physical activity can affect the measurements.^22^ Although this study detected a correlation between intensity of symptoms, as assessed by the translated questionnaire, and the volume of lower limbs, which could suggest inter-individual applications, this tool was developed for intra-individual comparison of symptoms to detect improvement or deterioration.

## CONCLUSIONS

The version of the visual analog lipedema symptoms questionnaire translated to, and culturally adapted for, Brazilian Portuguese is a practical instrument that is quick and easy to administer and can be used with our population for quantification of subjective data on lipedema. Further studies are needed to assess the instrument’s sensitivity as an aid to diagnosis of lipedema and its correlations with other aspects of lipedema.
