# Supplementary material for: Translation, cultural adaptation, and validation of a lipedema symptoms questionnaire
Source: J Vasc Bras. 2020 Aug 31;19:e20200049. doi: 10.1590/1677-5449.200049 (PMC8202216; doi:10.1590/1677-5449.200049)
Supplement: Translation

# Translation: Tradução, adaptação cultural e validação do questionário de avaliação sintomática do lipedema (QuASiL)

**Authors:** Alexandre Campos Moraes Amato, Fernando Campos Moraes Amato, Daniel Augusto Benitti, Ricardo Virgínio dos Santos

**Affiliations:** 1 Universidade de Santo Amaro – UNISA, Disciplina de Cirurgia Vascular, São Paulo, SP, Brasil.; 2 Amato - Instituto de Medicina Avançada, São Paulo, SP, Brasil.; 3 Medical Valens Center, Campinas, SP, Brasil.

**Keywords:** questionários, tradução, comparação transcultural, lipedema, obesidade, linfedema

## Abstract

**Contexto:**

O lipedema é caracterizado como uma deposição anormal de gordura em glúteos e pernas bilateralmente, que pode ser acompanhada por edema ortostático em mulheres. Foi publicado questionário de avaliação sintomática em lipedema em alemão e inglês para avaliação de evolução de tratamento.

**Objetivos:**

Tradução, adaptação cultural e validação do questionário de avaliação sintomática de lipedema para português brasileiro.

**Métodos:**

O processo consistiu em três traduções e duas retrotraduções realizadas por tradutores independentes, seguida da elaboração de versão consensual e adaptação conforme compreensão das pacientes. O questionário foi convertido em versão digital, aplicado em 56 voluntárias e em 154 pacientes de consultório vascular e correlacionado com volumetria por bioimpedância segmentar.

**Resultados:**

As 20 pacientes do pré-teste eram do sexo feminino e as questões foram compreendidas por pelo menos 90% das entrevistadas na fase final. Voluntárias mostraram compreensão de 96,4%, com tempo médio de resposta de 4 minutos. Em 154 pacientes, o volume dos membros mostrou correlação positiva com a intensidade dos sintomas avaliada pelo questionário traduzido e correlação fraca e moderada em apenas 3 perguntas, num total de 15 com nível de escolaridade.

**Conclusões:**

A versão traduzida e adaptada culturalmente para o português do Brasil do questionário de avaliação sintomática do lipedema (QuASiL) é um instrumento prático, de fácil e rápida aplicação, que pode ser utilizado em nossa população. Ainda são necessários estudos adicionais para avaliar a sensibilidade do instrumento para o auxílio no diagnóstico do lipedema.

## INTRODUÇÃO

O lipedema foi descrito pela primeira vez em 1940 pelos doutores Edgar Van Nuys Allen e Edgar Alphonso Hines Jr. na *Mayo Clinic* e foi caracterizado como uma deposição anormal de gordura em glúteos e pernas bilateralmente, que pode ser acompanhada por edema ortostático^1^^,^^2^ em mulheres. A fisiopatologia e a epidemiologia do lipedema é até hoje pouco compreendida, embora sejam sugeridos seu aspecto genético e sua influência hormonal em sintomas inflamatórios cíclicos^3^. Sendo assim, apesar de condição distinta, é frequentemente confundido com doenças mais frequentemente diagnosticadas, como a obesidade e o linfedema^4^^,^^5^. O diagnóstico é clínico e é tipicamente definido pela desproporção simétrica do acúmulo de gordura nas extremidades inferiores com queixas de edema ortostático^4^, frequentemente acompanhada por dor em peso, cansaço, tensão ou de difícil caracterização; constante ou ao toque nas áreas mais afetadas; com intensidade variável e sem irradiação. Os pés são poupados desse aumento de tamanho, exceto no estágio avançado de lipolinfedema, no qual o edema do pé ocorre secundariamente à insuficiência linfática inexistente em estágios anteriores^6^^,^^7^. O edema que poupa os pés é sinal importante para distinguir o lipedema da obesidade comum. A parte de cima do corpo (tronco) é poupada até os estágios mais avançados da doença, embora existam alguns subtipos de lipedema atípicos que podem variar do padrão esperado de acúmulo de gordura em extremidade inferior^8^^,^^9^. As áreas afetadas pelo lipedema frequentemente apresentam hematomas, dor e aumento de sensibilidade, acompanhados por queixas sistêmicas de fadiga e diminuição de condição física e força muscular. O início dos sintomas frequentemente começa durante a puberdade ou a jovem adulta, embora algumas pacientes possam iniciar até mais tardiamente^4^. Medidas conservadoras das estimativas da prevalência de lipedema na população em geral variam de 0,06 a 10%^4^.

Um questionário específico para o lipedema, sem nome, foi elaborado na Alemanha para avaliação sintomática pré e pós-operatória de lipedema e publicado por Rapprich et al.^10^ em alemão e inglês^11^. O questionário foi baseado no questionário de qualidade de vida para pacientes com doenças linfáticas^12^ e adaptado para incluir quinze critérios autoavaliados por escala analógica de 0 a 10. O questionário, baseado em avaliação de qualidade de vida, pode ser considerado uma escala sintomática do lipedema e não foi validado para uso como critério diagnóstico.

O questionário original é interpretado como intensidade dos sintomas por meio de uma pontuação derivada de avaliação analógica e visual, que pode variar de 0 a 150, sendo que 0 representa nenhuma queixa sintomática e 150, sintomas com o maior impacto em qualidade de vida. Tendo em vista a rara disponibilidade de instrumentos objetivos para avaliação de lipedema no Brasil e no mundo e que dados referentes à qualidade de vida são importantes para a seleção e interpretação da evolução clínica, justifica-se este estudo tendo como objetivo traduzir para a língua portuguesa e adaptar culturalmente para a população brasileira o questionário, além de validá-lo na prática clínica.

## MÉTODO

O presente estudo segue as normas do Conselho Nacional de Saúde, referentes à resolução n˚ 466/12 sobre pesquisa envolvendo seres humanos. Também segue a declaração de Helsinki e foi aprovado pelo Comitê de Ética em Pesquisa da Plataforma Brasil sob protocolo CAAE: 09590919.6.0000.0081.

### Método de tradução e adaptação cultural

O processo de equivalência linguística iniciou-se por meio de contato com o autor original para definição de conceitos e autorização para utilização destes. A tradução e a adaptação cultural do instrumento foram feitas com base em diretrizes existentes^13^^,^^14^, consistindo em tradução, retrotradução, revisão por painel de especialistas e adaptação cultural (Figura 1).

**Figura 1:** Fluxo de tradução, adaptação e validação.

Os itens da versão original do questionário em inglês foram traduzidos inicialmente por um profissional de saúde, independente, brasileiro, com grande conhecimento da língua inglesa e que estava ciente dos objetivos da pesquisa. O questionário em alemão foi traduzido separadamente por dois tradutores com grande conhecimento da língua alemã, cientes dos objetivos da pesquisa. Optou-se por duas versões do original em alemão devido ao autor ser nativo alemão. Foi enfatizada a importância da tradução conceitual em detrimento de uma tradução literal. Foram, assim, obtidas as versões de número 1 a 3 em português brasileiro. As traduções 1 a 3 foram avaliadas e unidas em versão consensual por dois médicos cientes dos objetivos e especialistas em lipedema. A versão consensual foi vertida para o alemão por dois professores de alemão, independentes, nativos, que não participaram da etapa anterior e não tinham conhecimento dos objetivos nem dos conceitos apresentados no questionário. Em seguida, foi feita a comparação do instrumento original com as duas novas versões em alemão. Um painel de especialistas composto por dois médicos documentou e analisou as discrepâncias encontradas. Alguns tempos verbais e sentenças em português foram reescritos até se obter um consenso, gerando, assim, a versão 4 em português.

A versão 4 em português do questionário foi aplicada a um grupo de 10 indivíduos para avaliar o grau de compreensão das questões, sendo selecionadas aquelas que não apresentavam boa compreensão. O painel de especialistas avaliou esses itens e os adaptou com o mesmo conceito, porém de mais fácil compreensão, utilizando, inclusive, sugestões dos próprios respondentes, procurando não alterar a estrutura e as propriedades de avaliação dessas questões e gerando a versão 5 do questionário. Essa versão 5 (Tabela 1) foi aplicada a outro grupo de 10 indivíduos, selecionados aleatoriamente no mesmo local de atendimento, sendo sua equivalência cultural testada novamente até que todos os itens fossem compreendidos por 90% dos entrevistados.

**Tabela 1:** Versão final do Questionário de Avaliação Sintomática de Lipedema.

Questionário de Avaliação Sintomática do Lipedema (QuASiL)											
Nome:						Data:					
Gradue seus sintomas de 0 (não) a 10 (muito). Se o critério for variável, selecione a intensidade máxima que você sente.											
	0	1	2	3	4	5	6	7	8	9	10
As áreas afetadas são dolorosas? (0 não / 10 muito)											
As áreas afetadas são sensíveis ao toque ou à pressão? (0 não / 10 muito)											
Você tende a ter manchas roxas facilmente e frequentes nas pernas? (Hematomas, equimoses) (0 não / 10 muito)											
Você sente “pressão” ou “tensão” nas pernas? (0 não / 10 muito)											
Sente as pernas “quentes” ou sensação de “queimação”? (0 não / 10 muito)											
Sente suas pernas frias? (0 não / 10 muito)											
Tem câimbras musculares? (0 não / 10 muito frequente)											
Sente peso nas pernas? (0 não / 10 muito)											
Sente cansaço nas pernas? (0 não / 10 muito)											
Sente inchaço nas pernas? (0 não / 10 muito)											
Tem “irritações” na pele? (0 não / 10 muita)											
Sente coceira? (0 não / 10 muita)											
Tem dificuldade para caminhar? Alguma limitação de movimento? (0 não / 10 gravemente)											
Como a condição afeta sua qualidade de vida? (0 nada / 10 gravemente)											
Está satisfeita com a aparência das pernas? (Atenção: 0 muito satisfeita / 10 insatisfeita)											

A versão 5 do questionário foi convertida para versão digital on-line com *software* seguro e apropriado para desenvolvimento e análise de questionários (SurveyMonkey, San Mateo, California) e foi aplicada em 56 voluntárias portadoras ou não de lipedema diagnosticado.

### Aplicação dos questionários

Os questionários foram aplicados individualmente, de junho a setembro de 2019, sendo os pacientes abordados previamente a consulta médica, explicados os objetivos e o conteúdo do questionário. Quando a pessoa preenchia os critérios de inclusão do estudo e tinha disponibilidade de tempo para responder imediatamente aos questionários, foi pedido o consentimento e colaboração para participar da pesquisa.

A técnica de amostragem foi não probabilística por conveniência, sendo que os sujeitos foram selecionados em ambulatório de cirurgia vascular. Os pacientes foram, então, submetidos a anamnese e exame físico não relacionados à pesquisa e exame de bioimpedância, e foram seguidos os critérios de inclusão e exclusão.

Foram incluídas mulheres, com idade maior que 18 anos, atendidas por qualquer queixa. Foram excluídos indivíduos do sexo masculino e aqueles que não assinaram termo de consentimento, que tinham condições arteriais ou venosas graves associadas e que não falassem ou compreendessem a língua portuguesa.

Na fase de validação on-line do questionário, voluntárias de grupo específico de portadoras de lipedema se propuseram a responder ao questionário de forma informatizada, em março de 2020, sem auxílio externo e preenchendo todos os dados de forma livre.

O estudo teve como objetivos primários a tradução e a adaptação cultural do questionário. Secundariamente, foi avaliada a correlação do escore de sintomas com variáveis indiretas da bioimpedância segmentar.

### Bioimpedância

A avaliação da composição corporal segmentar foi realizada com recurso de uma balança de bioimpedância digital multiespectral, na qual a resistência e a reactância corporal foram medidas (Tanita, BC-601, Illinois, USA). As medidas fornecidas pela bioimpedância [altura, peso, índice de massa corporal (IMC)] foram automaticamente copiadas em formulário próprio e outras variáveis foram inseridas em *software* criado especialmente para esse propósito, onde foi calculada medida de volume de membro inferior direito (MID) e esquerdo (MIE), individualmente, considerando a densidade gordura 0,9196 g/mL e músculo, 1,06 g/mL^15^^–^ 17.

### Análise estatística

Após verificação manual da consistência dos dados, foi feita a análise estatística. O método estatístico utilizado foi descritivo e de frequências. A correlação entre variáveis do questionário foi avaliada por correlação de Spearman e teste de Shapiro-Wilk. A relação entre o volume do membro e a intensidade dos sintomas no questionário foi avaliada pelo coeficiente de correlação de Pearson. A análise estatística foi realizada com auxílio do teste *t* de Student, teste de uniformidade de Kolmogorov-Smirnov e teste de Mann-Whitney. Assumimos, para as correlações, um nível de significância estatística de 0,05%. Os *softwares* utilizados para análise dos dados foram o Excel (Microsoft, Redmond, Washington, EUA) e Wizard 1.9.40 (Evan Miller, Chicago, IL, EUA).

## RESULTADOS

Vinte indivíduos participaram da fase de avaliação da adaptação cultural. Nenhuma questão foi considerada como não aplicável. As questões foram compreendidas por pelo menos de 90% dos entrevistados e elas foram modificadas e reaplicadas até que todos os itens apresentassem um nível de compreensão acima de 90%. A versão 5 final, aplicada on-line na população voluntária (Tabela 2), mostrou compreensão total de 96,4% da população estudada, com tempo médio de resposta de 4 minutos. Houve correlação moderada negativa entre a pergunta “Sente peso nas pernas?” e o grau de escolaridade (Spearman ρ -0,316, p = 0,018), correlação fraca negativa entre a pergunta “Sente cansaço nas pernas?” e o nível de escolaridade (Spearman ρ -0,292, p = 0,029) e correlação moderada negativa entre a pergunta “Está satisfeita com a aparência das pernas?” e o nível de escolaridade (Spearman ρ -0,309, p = 0,02). A somatória dos resultados não foi normal e uniformemente distribuída (Shapiro-Wilk z = 2,688, p = 0,004; Kolmogorov-Smirnov D = 0,35, p < 0,001), não apresenta correlação com peso, IMC ou mesmo grau de escolaridade, mas apresenta correlação fraca negativa com a idade (Pearson r = -0,280, p = 0,037). Foi acrescentada escala colorida para facilitar a compreensão de intensidade.

**Tabela 2:** Características da população estudada na validação on-line da versão 5.

**Característica do paciente**	**Média (mínimo–máximo ou IC)**
Voluntárias	56
Idade	38,69 (22–67)
Peso	79,46 (50–125)
Altura	1,62 (1,5–1,76)
IMC	30,10 (20,81–45,91)
Diagnóstico de lipedema prévio	71,4% (IC95% 58,5–81,6)
Diagnóstico de varizes prévio	73,2% (IC95% 60,4–83)
Grau de escolaridade	
Ensino superior completo	35,7%
Pós-graduação	35,7%
Ensino médio completo	12,5%
Ensino superior incompleto	12,5%
Ensino técnico	3,6%
Compreenderam integralmente o questionário	96,4% (IC95% 87,9–99)
Sugestões de mudança	Nenhuma
Demografia (estado)	
São Paulo	60%
Paraná	14,5%
Minas Gerais	7,3%
Rio de Janeiro	5,5%
Mato Grosso do Sul	3,6%
Rio Grande do Sul	3,6%
Santa Catarina	3,6%
Rio Grande do Norte	1,8%

IC = intervalo de confiança; IMC = índice de massa corporal.

A amostra foi constituída de 154 pacientes, que foram abordadas em ambulatório de cirurgia vascular (Tabela 3), independente do diagnóstico, realizaram bioimpedância com avaliação segmentar corporal e responderam ao questionário de avaliação sintomática de lipedema.

**Tabela 3:** Características da população estudada na validação prática.

**Características do paciente**	**Média (mínimo–máximo)**
Pacientes	154
Idade	43,74 anos (19–79)
Altura	163,86 cm (139–183)
Peso	79,612 kg (52,1–130,3)
IMC	29,657 kg/cm^2^ (20,6–45,6)
Volume MID	13.627,22 cm^3^ (9.051,57–24.819,61)
Volume MIE	13.448,379 cm^3^ (8.885,915–24.757,54)
Escore sintomas	79,091 (29–148)

IMC: Índice de Massa corpórea; MID: Membro inferior direito; MIE: membro inferior esquerdo.

O volume dos membros direitos e esquerdo possuem médias e medianas semelhantes (teste *t*, p = 0,627; teste de Mann-Whitney, p = 0,543) e ambas variáveis, analisadas individualmente pelo teste de Kolmogorov-Smirnov, não estão uniformemente distribuídas, p < 0,001(Figura 2).

**Figura 2:** Distribuição do volume (mL) dos membros e médias estimadas.

O volume do MID possui correlação fraca positiva com a intensidade dos sintomas avaliado pelo questionário traduzido (correlação de Pearson r = 0,186, p = 0,034) e o volume do MIE também possui correlação fraca positiva com a intensidade dos sintomas (correlação de Pearson r = 0,183, p = 0,037) (Figura 3).

**Figura 3:** Intensidade dos sintomas no questionário de avaliação (somatória de pontos) correlacionada com o volume (mL) do membro inferior direito (MID) e membro inferior esquerdo (MIE).

## DISCUSSÃO

Existe muita confusão e subdiagnóstico do lipedema devido à falta de um teste de laboratório ou um teste genético definitivo e fácil, associada à falta de familiaridade médica com os critérios de diagnóstico para o lipedema^18^. O lipedema é mascarado por outras condições e comorbidades, como linfedema, desproporção fisiológica do formato do corpo, lipohipertrofia, obesidade ginecoide, que podem contribuir para essa confusão e levar a um diagnóstico errado ou a um subdiagnóstico^19^. O diagnóstico errado em pacientes com lipedema é preocupante, pois atrasa o tratamento da doença e permite a sua progressão^18^. Até 50% dos pacientes com lipedema também estão em sobrepeso ou são obesos, sendo que a associação de comorbidades dificulta o diagnóstico, mas não impede que ele seja feito, pois há distinções importantes entre os cuidados para os pacientes com obesidade comum e lipedema.^19^

Sintomas inflamatórios do lipedema determinam a qualidade de vida do paciente^20^, e o paciente pode apresentar períodos de melhora e piora durante a vida. Atualmente não há possibilidade de acompanhar a evolução sintomática, melhora ou piora, a partir de exames laboratoriais ou de imagem no lipedema. Portanto, o questionário traduzido é uma ferramenta útil para avaliar o grau de comprometimento sintomático que o paciente se encontra e a evolução posterior, comparando-a consigo mesma.

Medir a influência na qualidade de vida da doença lipedema ainda é tarefa difícil, pois os sintomas e queixas são subjetivos e confundem-se com outras doenças muito frequentes no consultório vascular, como a insuficiência venosa crônica, obesidade e linfedema. Apesar de o assunto ter recebido atenção nos últimos anos e de já existirem vários instrumentos genéricos e específicos para realizar avaliação da qualidade de vida, tais instrumentos, na sua grande maioria, avaliam apenas os diagnósticos diferenciais, como insuficiência venosa, obesidade e linfedema. Por isso, é importante desenvolver e validar instrumentos que possam avaliar o impacto do lipedema na qualidade de vida e, se possível, auxiliar no diagnóstico definitivo.

Após a sequência de tradução, revisão, retrotradução e aplicação em população distinta do questionário, a versão final mostrou-se altamente compreensível pela população estudada (96,4%), com correlação fraca e moderada com nível de escolaridade em apenas 3 perguntas. Correlação não implica causalidade^21^. Devemos ressaltar que a pergunta “Está satisfeita com a aparência das pernas?” possui escala inversa para facilidade de compreensão e padronização do questionário, ou seja, o incômodo com a aparência das pernas é maior com menor escolaridade. Não houve palavras não compreendidas. A correlação inversa da somatória dos sintomas com a idade pode sugerir melhora dos sintomas ou mesmo aceitação dos sintomas pela paciente.

A bioimpedância segmentar aplicada é um método de fácil emprego e de alta reprodutibilidade, embora fatores como posição do indivíduo, posição das manoplas, temperatura ambiente, nível de hidratação e atividade física possam afetar essa medida^22^. Embora o presente trabalho tenha evidenciado correlação entre a intensidade dos sintomas, avaliada pelo questionário traduzido, com o volume dos membros inferiores, que pode sugerir utilidade interindividual, a ferramenta foi desenvolvida para comparação sintomática intraindividual de melhora ou piora dos sintomas.

## CONCLUSÃO

A versão do questionário visual analógico de sintomas do lipedema traduzida e adaptada culturalmente para o português do Brasil é um instrumento prático, de fácil e rápida aplicação, que pode ser utilizado na nossa população para quantificação de dados subjetivos referentes ao lipedema. Ainda são necessários estudos adicionais para avaliar a sensibilidade do instrumento para o auxílio diagnóstico do lipedema e correlação com outros aspectos do lipedema.
